# Persistence of Infectivity of Different Enteroviruses on a Surrogate Fomite: Correlation with Clinical Case Incidence

**DOI:** 10.3390/pathogens14080721

**Published:** 2025-07-22

**Authors:** Charles P. Gerba, M. Khalid Ijaz, Raymond W. Nims, Stephanie A. Boone

**Affiliations:** 1 Department of Environmental Science, University of Arizona, Tucson, AZ 85721, USA; sboone@arizona.edu; 2 Global Research and Development for Lysol and Dettol, Reckitt Benckiser LLC, Montvale, NJ 07645, USA; 3 Syner-G BioPharma, Boulder, CO 80301, USA; ray.nims@synergbiopharma.com

**Keywords:** echoviruses, enteroviruses, environmental transmission, fomites, persistence, poliovirus

## Abstract

Enteroviruses of the *Picornaviridae* family are transmitted primarily by the fecal–oral route. Transmission may occur following hand contact with contaminated fomites and subsequent ingestion of virus conveyed to the mouth by the contaminated hand. The persistence of these viruses on fomites likely plays a role in this transmission scenario. Six echoviruses (1, 2, 3, 5, 6, and 7) that cause frequently reported clinical cases in the United States were studied, along with poliovirus type 1 vaccine strain LSc-2ab. The infectivity half-lives of the enteroviruses deposited on vinyl tile coupons in a 10% fecal solution ranged from 1.7 to 12.6 h. The echovirus serotypes most commonly associated with reported infections persisted longer on the vinyl tiles than the less commonly reported types. This increased persistence on surfaces may favor the transmission of these echoviruses through the fecal–oral route. These results inform the future selection of appropriate model enteroviruses for challenging newly formulated and eco-friendly disinfectants or other strategies in infection prevention and control for enteroviruses.

## 1. Introduction

The *Enterovirus* genus of the *Picornaviridae* family includes 10 species of true enteroviruses and three species of rhinoviruses (the latter being respiratory viruses). Among these, the true enteroviruses infect the intestinal mucosa and cause local symptoms such as gastroenteritis, and symptoms at more distant sites such as meningitis, pancreatitis, and paralysis. The true enteroviruses include enteroviruses 68 and 71, poliovirus, coxsackie A and B viruses, and numerous echoviruses [1,2,3]. The picornaviruses are small (~30 nm), non-enveloped, single-stranded RNA viruses [2,3]. The enteroviruses are primarily transmitted by the fecal–oral route, and transmission frequently involves contact with contaminated feces, food, or water [3]. Transmission may occur following hand contract with enterovirus-contaminated fomites (environmental high-touch surfaces) and subsequent ingestion of virus conveyed to the mouth by the contaminated hand.

Fomite transmission of enteroviruses is well documented and is thought to play a significant role in the spread of these viruses [3]. The survival time (duration of persistence of infectivity on a fomite once contaminated with the virus) in the environment is likely to play a critical role in the potential for transmission of enteroviruses according to this scenario. To confirm this hypothesis, we have assessed the persistence at room temperature and 40–60% relative humidity (RH) of echoviruses 1, 2, 3, 5, 6, and 7 and poliovirus 1 on a vinyl surface and have related the determined persistence half-lives to the frequencies of reporting of cases for each echovirus published by the United States Centers for Disease Control and Prevention (U.S. CDC) [2,4,5,6].

Echoviruses were selected for this study because they cause a wide variety of mild and serious illnesses and because their ease of culturing and assay in cell culture facilitates detection of the viruses on surfaces. Poliovirus type 1 vaccine strain LSc-2ab (Sabin) was also included as a comparison as poliovirus (typically PV-1) has been used as a surrogate strain for previous environmental stability and standardized viral inactivation efficacy studies [7,8,9,10,11,12,13,14,15,16,17,18,19,20,21,22,23,24,25,26,27].

Echoviruses cause a wide variety of illnesses from minor febrile illness to severe, potentially life-threatening conditions (e.g., aseptic meningitis, encephalitis, paralysis, myocarditis) [1,3]. Individual serotypes have different temporal patterns of circulation in the environment and cause different clinical manifestations. Echoviruses 5–7 are among the most frequently reported enteroviruses reported in the United States [2,4,5,6]. In fact, from 1970 to 2005, echovirus 6 was the fifth most common enterovirus reported to the U.S. CDC (based on the numbers of cases reported with species identification performed) [2] and is still commonly isolated from clinical specimens [4,5,6]. The rankings of the different echovirus types reported have varied from year to year, but some types have been consistently reported [2,4,5,6].

Many factors impact the persistence of viruses on surfaces (fomites) including temperature, RH, drying rate, type of surface (hard, soft; porous, non-porous), and presence of virally associated pathophysiological bodily fluids (organic load) [16,28,29]. Generally, viruses survive longer on fomites in the presence of an organic load and on hard surfaces (stainless steel, vinyl) [16,28,29]. Transfer of pathogens to the hands upon touching a contaminated surface is facilitated when the surface is hard vs. soft [30].

In this study we evaluated the persistence of infectivity of the various enteroviruses suspended in an organic load (10% fecal solution) and deposited onto vinyl coupons held at room temperature. The infectivity decay half-lives for each enterovirus were calculated, compared with that of poliovirus-1 Sabin, and related to the frequency of reported enterovirus cases reported to the U.S. CDC.

## 2. Materials and Methods

The types of echoviruses for this study were selected based on the availability in our laboratory, the growth to sufficient titer in cell culture for infectious virus detection assays enabling the experimental work, and their abilities to cause reportable illness. The vaccine strain poliovirus type I (LSc-2ab) was included because data on the persistence of this virus on fomites and other environments are available in the primary literature. The sources for the viruses and the types of cell lines used for amplification and detection of the viruses, as well as the growth media used for the cell lines, are shown in Table 1. The buffalo green monkey cell line (BGM) was obtained from Daniel Dahling of the Biological Methods Branch of the Environmental Monitoring and Support Laboratory, U.S. Environmental Protection Agency, Cincinnati, OH, USA. The LLC-MK2 cells were obtained from the Baylor College of Medicine cell culture collection, Houston, TX, USA. All viral titration and detection assays were performed using the plaque-forming unit (PFU) method [31]. The cells were grown in Eagle’s Minimum Essential Medium supplemented with 10% fetal bovine serum, glutamine, vitamins, and non-essential amino acids. Viruses were propagated in host cells maintained in media without FBS.

The viruses were released from the infected cells by freeze–thawing and the resulting suspensions were subjected to centrifugation to remove cell debris. The amounts of the echoviruses added to the vinyl coupons varied from 4.98 log_10_ PFU to 3.53 log_10_ PFU, depending on the titers of the individual viruses obtained during amplification in cell culture. Poliovirus could be grown to a greater titer and 5.7 log_10_ PFU was added to the coupons. Viruses were added to a 10% suspension of human feces in distilled water and 1.0 mL aliquots of the resulting mixtures were applied to 1 × 1-inch cut vinyl tile coupons (carriers). The coupons were placed in a plastic beaker and held at room temperature (~27 °C). After 24 and 48 h, 3 mL of trypticase soy broth (Difco, BD, Franklin Lakes, NJ, USA) was added to each coupon and the coupons then were placed on a shake table for 15 min to elute the virus. The elution fluids were then assayed for infectious virus after the addition of antibiotics to suppress bacterial growth. Log reduction of the virus was determined by comparing the initial titers of the virus (after immediately adding the virus to the coupon at time zero and eluting the virus) to the titers obtained after 24 and 48 h. This approach negated any potential differences in elution efficiency between the various serotypes.

Infectivity decay half-lives for each enterovirus on the vinyl coupons at room temperature and 40–60% RH were calculated from plots of the log_10_ reductions in recovered titer vs. time on stability, using the formula t_1/2_ (h) = (0.301/slope) (see Supplementary Materials Figures S1–S3 for stability plots). All assays were conducted in duplicate for all time points. The 95% confidence intervals for the decay half-lives were obtained by calculating the positive and negative 95% intervals for the decay slopes (containing three points) and dividing 0.301 by these. 

## 3. Results

The six echoviruses evaluated in this stability study differ with respect to the frequencies with which they caused illnesses reported to the U.S. CDC during the reporting period of 1970–2005 [2] (Table 2). For instance, echovirus 6 represented 6.1% of all enterovirus cases reported, while echoviruses 1 and 2 represented less than 1% of the reported cases. The survival (persistence of infectivity) of the six echo and polio viruses on the vinyl coupons at room temperature and 40–60% RH is shown in Table 2. Each of the enteroviruses, with the exception of echovirus 1, remained infectious for at least 24 h on the vinyl coupons. No infectious echovirus 1 was recovered at 24 h. Of the various enteroviruses, only echoviruses 5 and 6 and poliovirus remained infectious on the vinyl coupons after 48 h. In this study, the various enterovirus stocks were not able to be amplified to the same initial titers, and this accounts for differences in the maximal log_10_ reductions observed for the echoviruses 1, 2, 3, 6, and 7 and poliovirus 1 during the 24 h and 48 h stability time intervals.

**Figure 1:**
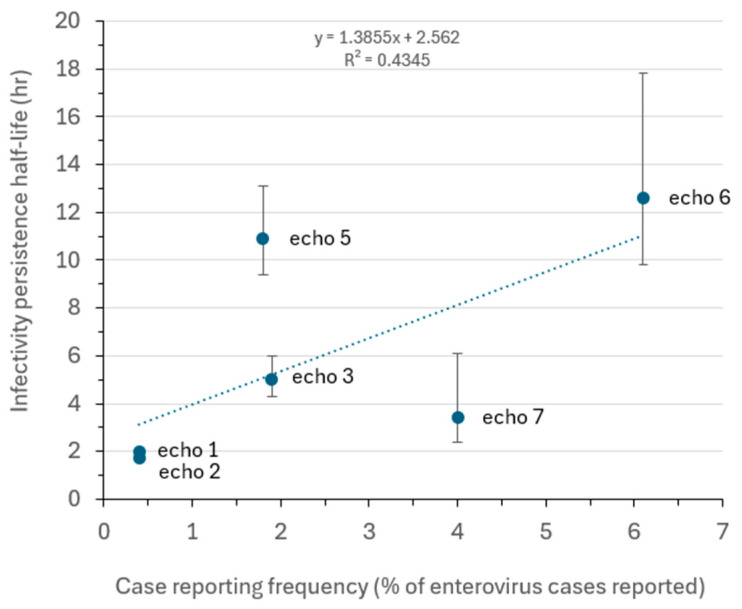
Relationship between persistence half-life (h) for echoviruses 1, 2, 3, 5, 6, and 7 and % of enterovirus cases reported to the U.S. CDC between 1970 and 2005. Abbreviations used: echo, echovirus; h, hours. The linear regression line is shown, along with the line equation. The correlation coefficient (*r* value) is 0.659 (*p* < 0.05, by one-tailed *t*-test). The error bars indicate the 95% confidence intervals for the determined half-lives.

Infectivity decay half-lives for each enterovirus on the vinyl coupons at room temperature and 40–60% RH are displayed in Table 2. These t_1/2_ values ranged from 12.6 h for echovirus 6 to <1.7 h for echovirus 2. The correlation between persistence half-life in h and percentage of enterovirus cases reported to the U.S. CDC between 1970 and 2005 is depicted in Figure 1. The correlation coefficient (*r*) of 0.659 indicates a moderate positive relationship.

## 4. Discussion

Echovirus 6 is one of the most common causes of infections attributed to enteroviruses in the United States [2,4,5,6]. In a study of virus infections among families from the early 1950s through 1970 in several major cities in the United States, this echovirus was found to be the most common echovirus infection [32]. This echovirus is still a leading cause of echovirus infections worldwide. The U.S. CDC Enterovirus Watch program has also reported its common occurrence. Echoviruses 3, 5, and 7 have been less commonly reported, but more so than echoviruses 1 and 2. For instance, no clinical cases of echoviruses 1 and 2 have been reported to the U.S. CDC since 2005 [4,5,6]. This infrequent reporting may reflect either milder illness caused by echoviruses 1 and 2 or a greater number of asymptomatic cases for these types. Even given these possibilities, there appears to be a relationship between the persistence half-lives of these echoviruses on the studied fomite and the incidence of reported cases. Such a relationship may be important in understanding the potential for the environmental transmission of these viruses and strategies for mitigating risk of their transmission through the fecal–oral route via contaminated high-touch environmental surfaces in home and community settings, including healthcare facilities.

Persistence of viruses on inanimate surfaces is evaluated because such persistence data inform the role of the indirect transmission pathway for viruses [33,34,35,36]. As mentioned previously, indirect transmission may occur following hand contract with fecally contaminated fomites (environmental high-touch surfaces) and the subsequent ingestion of virus transferred from the contaminated hand to the mouth. The longer the duration of time that a given virus remains infectious once deposited on a surface, the more opportunities there are for the virus to be transferred to a susceptible mucous membrane such as the oral or nasal mucosa and to cause an infection in that person. Several studies of the persistence of poliovirus have been reported. For instance, Mbithi et al. [10] found the persistence half-life of PV-1 Sabin deposited on steel carriers in a 10% fecal solution to be 2 or 7 h at 20 °C/25% RH or 20 °C/95% RH, respectively. Abad et al. [11] found the persistence half-life of PV-1/LSc 2ab deposited in a 20% fecal solution on china, aluminum, or latex carriers to be <4.3 h at 20 °C/50% RH. These values are similar to the value obtained in our study for PV-1 deposited in a 10% fecal solution on vinyl tiles (5.7 h, 95% confidence interval = 5.5 to 5.9 h) at room temperature (~27 °C) and 40–60% relative humidity. On the other hand, Tuladhar et al. [14] found the persistence half-life of PV-1 Sabin deposited on glass or plastic carriers to be ~73 or ~72 h, respectively, at room temperature. In that study, the presence or absence of organic material in the stability matrix and the relative humidity during the persistence evaluation were not specified. It is not clear exactly why the persistence half-lives reported by this group were so different from those found in our study and those mentioned above.

Published data on the comparative persistence of different echoviruses on hard non-porous surfaces were not identified during our search of the primary literature. Sittikul and coworkers [36] found the persistence half-lives of coxsackie virus A16 strain TH/MUMT-1/2015 and enterovirus A71 strain TH/MU-1/2015 deposited onto wood, plastic, or steel carriers in a matrix of culture medium containing 15% fetal bovine serum to range from 0.7 to 1.8 h or 1.6 to 3.5 h, respectively. Mocé-Livinia observed that echovirus 6 and PV-1 strain LSc 2ab displayed similar persistence half-lives (5.8 and 6.0 h, respectively) on a porous surface (cellulose membranes) at room temperature and 20% RH [12].

Poliovirus type 1 has previously been stipulated as a challenge virus in ASTM and EN disinfectant efficacy testing standards [17,18,19,20,21,22,23,24], and as a tier 1 surrogate for emerging pathogens by the U.S. Environmental Protection Agency (EPA) [25] and European guidance documents [26,27]. As a result of the current global efforts to eradicate poliovirus [37], initiatives have been implemented to reduce or safely contain existing stocks of poliovirus globally [38,39]. This means that since 31 December 2022 the use of poliovirus as a challenge virus in disinfectant efficacy has essentially been restricted to poliovirus-essential facilities (although a legal framework does not exist within the United States for such restrictions as of 2024 [39]), and suitable substitute challenge viruses will need to be identified and utilized at any facilities that are not certified as poliovirus-essential facilities. Our stability results, measured empirically well before December 2022, suggest that the assessment of environmental interventions for mitigating risk of transmission of echoviruses and the development of new disinfectants might utilize echovirus 6 as a worst-case challenge enterovirus (i.e., enteric virus within the family *Picornaviridae*) going forward.

Some limitations of our study are that it involved a limited subset of the serotypes of the *Enterovirus* genus and did not take into account potential intra-serotype differences in persistence; it examined persistence of infectivity on a hard, non-porous vinyl surface only; it did not consider the various potential mechanisms leading to virus decay on surfaces; and the persistence data points were limited to 24 h and 48 h only. Other limitations include that reporting to the CDC by the states is voluntary, changes in detection methodology (e.g., cell culture/serology to molecular methods) may have occurred over time, and epidemiological patterns may have evolved over time [5].

Another aspect to be considered in selecting a model enterovirus for such studies is the comparative susceptibilities of these viruses to commonly used virucidal active ingredients and formulated microbicides. On the basis of the expected mechanisms of action of microbicides [40], one might assume the various enteroviruses to display roughly similar susceptibilities to the inactivating effects of microbicides. Despite this expectation, review of the viral inactivation literature has identified a number of intra-family differences in susceptibility for the picornaviruses [41]. From that review, it appears that the echoviruses, as a group, tend to be similarly susceptible, and in some cases less susceptible, than poliovirus to a number of microbicidal active ingredients. A fair amount of literature comparing enterovirus susceptibilities to a limited number of microbicidal actives (especially chlorine and glutaraldehyde) has been published in suspension [42,43,44,45,46] and surface inactivation studies [12,47], and in hand hygiene studies [48,49]. None of the published studies have evaluated echoviruses 5 or 6 and poliovirus side-by-side. What remains to be performed, therefore, are comparative studies of the susceptibilities of a variety of echoviruses, including especially the serotypes found to be most persistent in this study (echoviruses 5 and 6), or coxsackie viruses to a variety of commonly used microbicides, conducted side-by-side along with poliovirus. Such studies, taken together with our comparative persistence results, may then be used to justify the selection of the most appropriate enteroviruses as a replacement for poliovirus as a worst-case challenge enterovirus.

## 5. Conclusions

In summary, our results on the persistence of viral infectivity on a vinyl surface suggest that the assessment of environmental interventions for mitigating risk of transmission of echoviruses and the development of new disinfectants might utilize echovirus 6 as a worst-case challenge enterovirus (i.e., enteric virus within the family *Picornaviridae*) going forward.

## Figures and Tables

**Table 1:** Viruses and cell lines used for assay.

Virus (Strain) ^1^	Source	Cell Line Used for Titrations
Echovirus 1 (Farouk)	ATCC ^2^ VR-1808	BGM (Buffalo green monkey)
Echovirus 2 (Cornelis)	ATCC VR-1867	BGM (Buffalo green monkey)
Echovirus 3 (Morrisey)	ATCC VR-33	BGM (Buffalo green monkey)
Echovirus 5 (Noyce)	ATCC VR-35	LLC-MK2 (Lewis lung carcinoma—monkey kidney 2)
Echovirus 6 (D-1 [Cox])	ATCC VR-241 L	LLC-MK2 (Lewis lung carcinoma—monkey kidney 2)
Echovirus 7 (Wallace)	ATCC VR-37	BGM (Buffalo green monkey)
Poliovirus 1 (LSc-2ab)	BCM ^3^	BGM (Buffalo green monkey)

^1^ ICTV virus nomenclature. https://ictv.global/report/chapter/picornaviridae/picornaviridae/enterovirus (accessed on 18 July 2025). ^2^ American Type Culture Collection, Manassas, VA, USA. ^3^ Baylor College of Medicine, Department of Virology and Epidemiology, Houston, TX, USA. Culture Collection.

**Table 2:** Persistence at room temperature (~27 °C) and 40–60% relative humidity of enteroviruses suspended in 10% feces solution and deposited onto a vinyl surface.

Virus (Strain)	Incidence Rank of Enterovirus Cases Reported to U.S. CDC (1970–2005) ^1^	% of Enterovirus Cases Reported to the U.S. CDC (1970–2005) ^2^	Log_10_ Reduction in Titer After:		Infectivity Half-Life (h)
			24 h	48 h	
Echovirus 6	5	6.1	0.36	1.25	12.6
Echovirus 7	10	4.0	0.94	>4.80	3.4
Echovirus 3	14	1.9	1.14	>3.04	5.0
Echovirus 5	15	1.8	0.80	1.25	10.9
Echovirus 2	28	0.4	4.20	>4.20	<1.7
Echovirus 1	29–30	0.4	>3.53	>3.53	<2.0
Poliovirus 1 (LSc-2ab)	Vaccine strain—spreads among populations	-	1.32	2.52	5.7

^1^ Incidence rank data are from the U.S Centers for Disease Control and Prevention (U.S. CDC) [2], i.e., echovirus 6 was the fifth most common enterovirus infection reported to the U.S. CDC. ^2^ The enteroviruses are listed in order of the number of cases reported to the U.S. CDC from 1970—2005 [2]. Note: from 2019–2024—no echovirus 2 cases were reported to the U.S. CDC (i.e., echovirus 2 represented less than 1% of all enterovirus cases) [4,5,6]. This indicates that echovirus 2 remains to the present time a very infrequently reported serotype.

## Data Availability

All data are contained within the article and Supplementary Materials Figures S1–S3 (stability plots).

## Supplementary Materials

The following supporting information can be downloaded at: https://www.mdpi.com/article/10.3390/pathogens14080721/s1, Figure S1: Estimation of infectivity half-life on vinyl surface for echovirus 5 and echovirus 6; Figure S2: Estimation of infectivity half-life on vinyl surface for echovirus 7 and echovirus 3; Figure S3: Estimation of infectivity half-life on vinyl surface for poliovirus type 1, echovirus 1, and echovirus 2.
