# Supplementary material for: Persistence of Infectivity of Different Enteroviruses on a Surrogate Fomite: Correlation with Clinical Case Incidence
Source: Pathogens. 2025 Jul 22;14(8):721. doi: 10.3390/pathogens14080721 (PMC12388917; doi:10.3390/pathogens14080721)

Supplemental Material

Persistence of Infectivity of Different Enteroviruses on a Surrogate Fomite: Correlation with Clinical Case Incidence

Charles P. Gerba, M. Khalid Ijaz, Raymond W. Nims and Stephanie A. Boone

Figure S1. Estimation of infectivity half-life on vinyl surface for echovirus 5 and echovirus 6. The regression lines were forced through the origin. Infectivity half-life in hours (12.6 hr for echovirus 6; 10.9 hr for echovirus 5) was calculated as 0.301/slope.

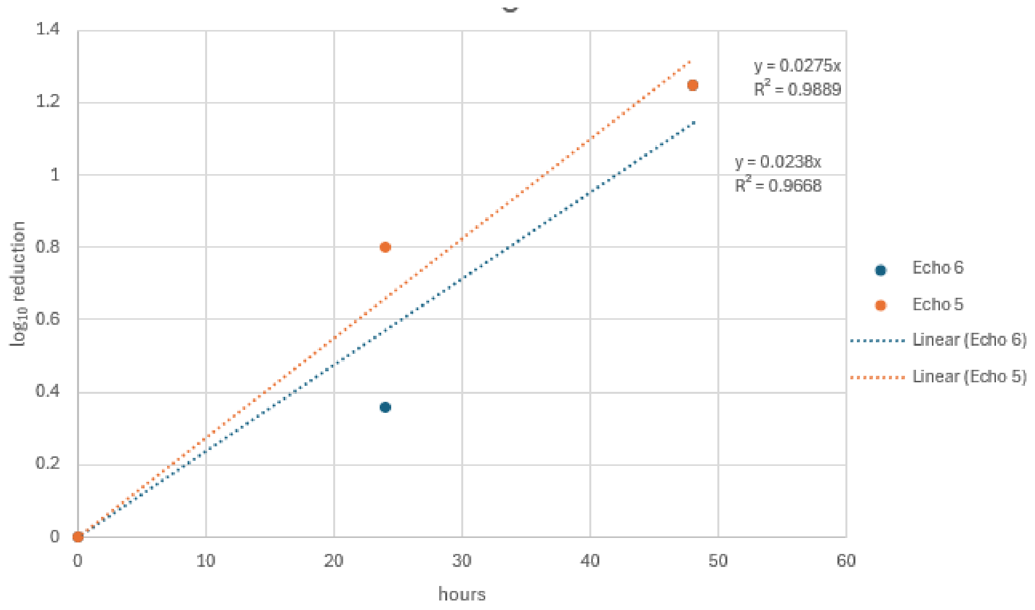

Figure S2. Estimation of infectivity half-life on vinyl surface for echovirus 7and echovirus 3. The regression lines were forced through the origin. Infectivity half-life in hours (3.4 hr for echovirus 7; 5.0 hr for echovirus 3) was calculated as 0.301/slope.

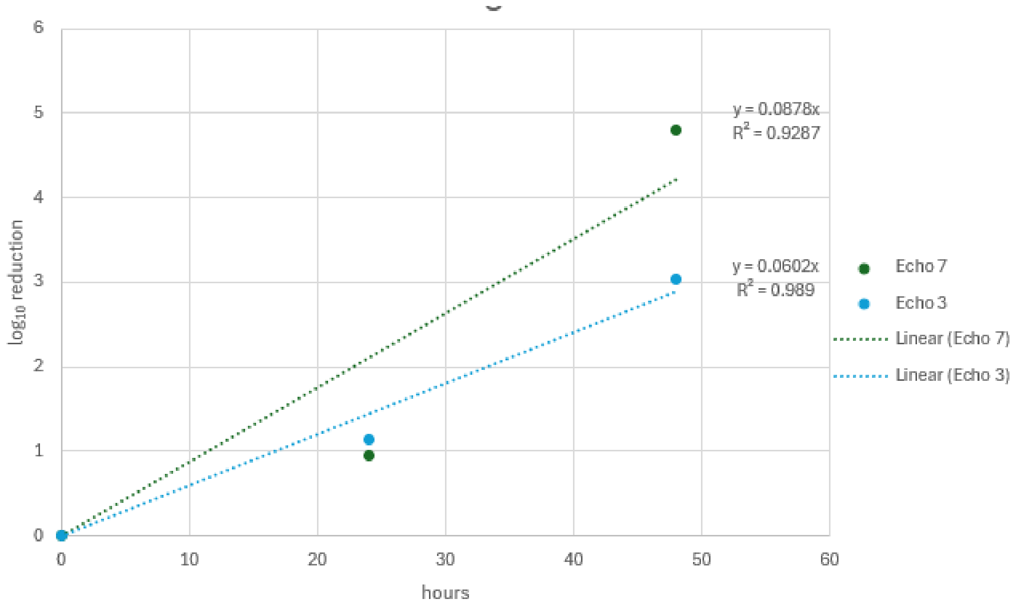

Figure S3. Estimation of infectivity half-life on vinyl surface for poliovirus type 1, echovirus 1, and echovirus 2. The regression lines were forced through the origin. The regression lines for echovirus 1 and echovirus 2 only include the 24-hr timepoint since inactivation was complete as of that point. Infectivity half-life in hours (5.7 hr for poliovirus type 1; <2.0 hr for echovirus 1; <1.7 hr for echovirus 2) was calculated as 0.301/slope.

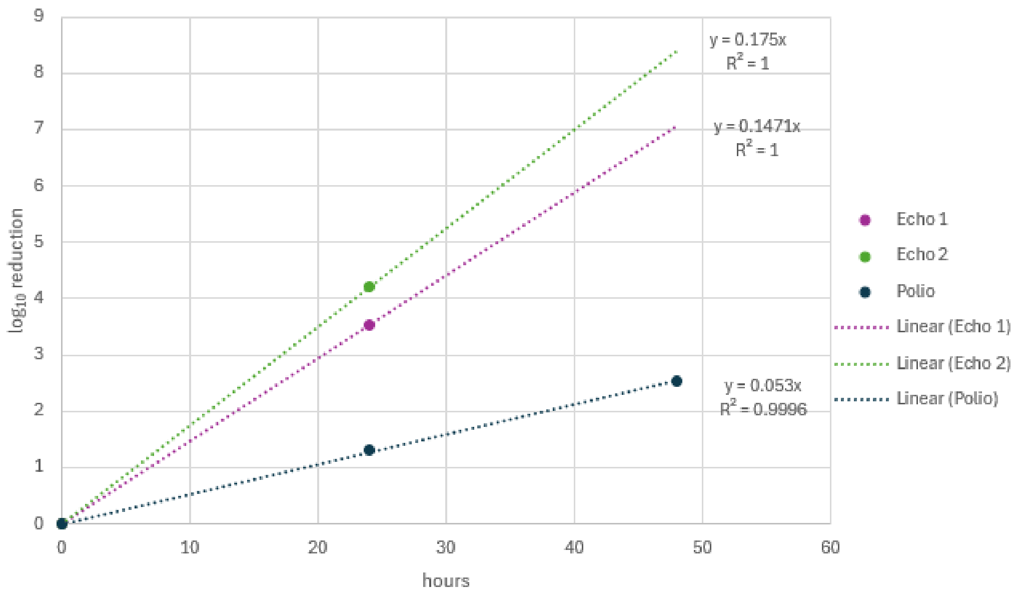

Supplement: Supplementary file 1 [file pathogens-14-00721-s001.zip › pathogens-3748674-supplementary.pdf]
